# Searching for the optimal measuring frequency in longitudinal studies -- an example utilizing short message service (SMS) to collect repeated measures among patients with low back pain

**DOI:** 10.1186/s12874-016-0221-4

**Published:** 2016-09-13

**Authors:** Iben Axén, Lennart Bodin

**Affiliations:** Institute of Environmental Medicine, Unit of Intervention and Implementation Research in Worker Health, Karolinska Institutet, Nobels väg 13, S-171 77 Stockholm, Sweden

**Keywords:** Short message service, Text messages, Repeated measures, Low back pain

## Abstract

**Background:**

Mobile technology has opened opportunities within health care and research to allow for frequent monitoring of patients. This has given rise to detailed longitudinal information and new insights concerning behaviour and development of conditions over time.

Responding to frequent questionnaires delivered through mobile technology has also shown good compliance, far exceeding that of traditional paper questionnaires. However, to optimize compliance, the burden on the subjects should be kept at a minimum.

In this study, the effect of using fewer data points compared to the full data set was examined, assuming that fewer measurements would lead to better compliance.

**Method:**

Weekly text-message responses for 6 months from subjects recovering from an episode of low back pain (LBP) were available for this secondary analysis. Most subjects showed a trajectory with an initial improvement and a steady state thereafter.

The data were originally used to subgroup (cluster) patients according to their pain trajectory. The resulting 4-cluster solution was compared with clusters obtained from five datasets with fewer data-points using Kappa agreement as well as inspection of estimated pain trajectories. Further, the relative risk of experiencing a day with bothersome pain was compared week by week to show the effects of discarding some weekly data.

**Results:**

One hundred twenty-nine subjects were included in this analysis. Using data from every other weekly measure had the highest agreement with the clusters from the full dataset, weighted Kappa = 0.823. However, the visual description of pain trajectories favoured using the first 18 weekly measurements to fully capture the phases of improvement and steady-state. The weekly relative risks were influenced by the pain trajectories and 18 weeks or every other weekly measure were the optimal designs, next to the full data set.

**Conclusions:**

A population recovering from an episode of LBP could be described using every other weekly measurement, an option which requires fewer weekly measures than measuring weekly for 18 weeks. However a higher measuring frequency might be needed in the beginning of a clinical course to fully map the pain trajectories.

## Background

The world has changed tremendously during the past decade in terms of communication. In high income countries most people own mobile phones [1], and the low income countries are not far behind [2–5]. As well as talking to and messaging each other, the phone is becoming a substitute for timetables, newspapers and calendars. Modern communication has also influenced the world of medicine. Mobile phone applications are available for different aspects of health monitoring [6, 7], fitness [8] and behavioural change [9]. Reminders are sent to patients enrolled in vaccination programs [10], monitoring of symptoms in chronic conditions is now possible [11–13] and adherence to medication use seems to improve with this technology [14]. Medical research is also catching on, and mobile phones are, for instance, used to monitor subjects after an intervention [15, 16] or are being used as integral parts of interventions [17].

The use of mobile communication enables unique insights into conditions that vary over time. It is known that many types of pain fluctuate during the day, week and over longer time periods. As frequent measurement is feasible with this new technology, the variability of the pain experience may be captured and studied [18, 19]. Contrary to diaries [20], time-stamping of data recordings is possible, which allows for recall bias to be assessed. Further, people carry their phone with them at work, at home and on vacations, rendering compliance unaffected by time and season [21]. Adding to the positive features of this method is also the cost. Generally, it is much cheaper to use mobile communication compared to paper questionnaires and ordinary mail services [22].

For any type of measurement, good compliance and minimal amount of missing data are essential features of accurate estimates. In many longitudinal surveys, compliance drops as time passes, rendering conclusions regarding the measured variable uncertain. However, studies using mobile phones as the data collection tool have reached good compliance [19, 21], even in long term follow ups and even among the young men [23] that usually drop out of studies. However, the burden to the participants should be kept at a minimum to optimize compliance.

Concerns have been raised that intense monitoring may have a reactive effect, that the attention towards a certain symptom or behaviour will influence the very item being studied [24]. Some studies have found no such effect [25, 26], and one study suggests that psychological states like anxiety and depression may actually decrease after a period of intense monitoring of pain [27].

It would be desirable to find the optimal number of measurements, i.e. the minimal number of data points to adequately describe the variability of the measure without losing detail. This needs to be balanced by optimal subject compliance, i.e. participants who keep answering throughout the study follow up without a reactive effect.

The aim of this study was to explore the optimal frequency for measuring pain repeatedly over 6 months. By comparing the pain trajectories formed by the full dataset of 26 weekly measures with those utilizing only parts of the data, it was possible to assess the effect of using fewer data points on the pain trajectory. As the compliance for the full dataset (26 weekly test messages) was known, it was assumed that fewer measuring points would render compliance at this level or higher as the burden on the participants would decrease.

## Method

This was a secondary analysis of data collected in a longitudinal observational study [21].

In short, the source population consisted of 244 patients with non-specific low back pain (LBP) who consulted a chiropractor in Sweden for this problem. Therefore, most of the subjects were experiencing a pain episode at baseline. Inclusion criteria in the study were LBP with or without leg pain, working age, having access to a mobile phone, knowledge of how to use the text message function of their phone as well as fluency in Swedish. Exclusion criteria were red flags (serious pathology), pregnancy and specific LBP (such as disc herniation). Demographic and baseline data are shown in Table 1.

**Table 1:** The baseline characteristics of the original full data set and those of the study sample

Variable	Original study *N =* 244	This study *N =* 129
Age, median (IQR)	43 (35–53)	46 (36–54)
Gender, % female	48	50
Pain, VAS, 1–10, median (IQR)	4.0 (3–6)	4.0 (3–6)
Leg pain, %	49.3	47.2
Duration of LBP > 30 days %	58.4	55.0
Health-related quality of life:		
EQ-5D mean (SD)	0.715 (0.21)	0.734 (0.20)
General Health median (IQR)	2 (2–3)	2 (2–3)

The participants received a text message every Sunday for 26 weeks with a question about their LBP: “*How many days during the previous week has your low back pain been bothersome, (i.e. affected your daily activities or routines)? Please answer with a number between 0 and 7”.* The text messages were sent through a system called SMS Track [28], and the text message replies were instantly recorded in a data file on line, suitable for analysis. The primary outcome, the recorded weekly value, is the Number of Bothersome Days (NBD).

The weekly text message data were used to map the individual pain trajectories of the respondents and to group the patients with similar courses together in a cluster analysis [29]. One of the clusters had a rather stable course over time and the remaining three showed improvement of varying speed, after which a “steady state” ensued [29]. Therefore, the data in this study stem from a patient cohort where the individuals sought care when in pain and map a clinical course in which the majority of patients improve.

To explore the effects of varying frequencies of follow-up, a complete dataset was needed in this secondary analysis to be used as a reference dataset for comparisons to follow. Thus, data imputation was used in all missing cells for NBD. To ensure solid estimates, subjects had to have a minimum of 24 out of 26 weekly text message replies, as well as a full set of the following baseline variables: Age, sex, duration of LBP and health-related quality of life (measured through the Euro Qol 5-dimentions (EQ-5D) and a single item “How would you rate your health?” with answer options ranging from Excellent [1] to Poor [5]). Imputation for missing values of the weekly NBD was done by using the mean value of NBDs from the observations closest in time, before and after the missing data point. Further, because the spline regression analysis requires some variability in the individual data, 4 subjects with a constant reply (e.g. NBD = 7 for all weeks) were removed. Thus, data from 129 subjects from the original study were used in this analysis.

To explore the effects of varying frequencies of measuring, 6 different options were tested. These frequency options were based on the previous results, namely that some groups of individuals seem to move extremely quickly towards recovery (in a matter of 3 weeks), some relatively quick (during the first 8 weeks), others slower (during weeks 13 to 18) and some did not show much improvement at all. Thus the frequency options explored were;

A) all data (=26 weekly measures) which served as a reference,
B) the first 8 weekly measures only,
C) the first 13 weekly measures only,
D) the first 8 weekly measures, then every fourth weekly measure supplemented with the last weeks measure, week 26, thus 13 weekly measures in all,
E) every second weekly measure (13 weekly measures evenly distributed across the study period, also including the last week (week 26)), thus 14 weekly measures, and
F) the first 18 weekly measures.

Each of the 6 frequency options were analysed as in the original article [29], using a cluster analysis based on parameters obtained from spline regressions. This was a person-oriented analysis [30], of individual pain trajectories, implying that the analysis concerns the pain course of each one of the individuals, regardless baseline variables. To estimate each individual course, the spline regression was used to derive two regression lines (to describe the early and the late course of the pain experience, respectively). The point of intersection (the knot) between these two lines was also estimated along with the parameters for slopes and intercepts of the two regressions. For each individual the spline regression resulted in four unique parameters and these parameters were then used in two supplementary clustering algorithms to explore a potential clustering of the individuals with similar trajectories.

The first clustering algorithm was an hierarchical cluster analysis (Ward’s method) to identify a preliminary set of clusters, each with specific characteristics [31]. These clusters were then used as the start for the second clustering procedure (K-means clustering), to consolidate the cluster formation and obtain the optimal number of clusters. This optimal number was noted according to the Calinski-Harabasz criterion [31].

Using this criterion, a four cluster solution was found to be optimal in the reference data set A), in line with the original reference [29]. Our first evaluation of clusters therefore aimed at comparing this four-cluster solution for the reference data set with four-cluster solutions from each frequency option B-F, using the same approach with Ward’s and the K-means methods. To obtain a quantification of the agreement between clusters from the reference data set A) and the tested frequency options B)-F) Kappa and Weighted Kappa was used [32]. Landis and Koch [33] give indicative values of Kappa to describe the degree of agreement with Kappa above 0.75 as excellent agreement, values below 0.40 as poor agreement and values in the range 0.40–0.75 as fair to good agreement. This first evaluation compared the frequency options on the assumption that based on previous studies, a four cluster solution is the relevant classification of individuals within this particular population.

A second evaluation applied a more exploratory approach where the assumption of a four-cluster solution was relaxed and different numbers of clusters were analysed, using the Calinski-Harabasz criterion to determine the optimal number of clusters. Thus, the reference data was as before confined to its optimal four cluster solution but for the frequency options B)-F) a non-restricted search for optimal numbers of clusters was used. In this case the Kappa criterion was not suitable as it requires comparisons between equal numbers of clusters. Therefore a graphical comparison of trajectories was used. The trajectories were derived as the average trajectories for each cluster, using the available data under the frequency option, and also extending the estimated trajectories to cover the whole period up to week 26.

In a different analytical approach a generalized linear model (GLM) was used to estimate group differences in the risk for the outcome event “bothersome day” separately for each week, from 1 to 26. The reported outcome variable was the NBD, for each week. The specified distribution for NBD was a binomial distribution with a fixed number of days (that is, 7 days), and NBD varied between 0 and 7 depending on the actual number of bothersome days reported during each week examined. If a group factor is introduced in the GLM a group comparison with respect to the risk of a bothersome day can be estimated, and by using a logarithmic link function in GLM we obtained estimates of the Relative Risk (RR) for each one of the 26 weeks.

For the purpose of this comparison the chosen group factor was duration of pain the previous year, as this was the only variable that consistently showed a predictive ability for LBP over a 6 month follow-up in a previous study [34]. The variable was dichotomized into ≤ 30 days vs > 30 days of pain the previous year. The outcome parameter, RR, thus estimates the relative risk for a bothersome day comparing pain > 30 days as the index category and pain ≤ 30 days as the reference category.

Ethics permission was granted by the Karolinska Institutet; 2007/1458–31/4. All participants signed informed consent forms.

## Results

In total, 98 points of data were imputed (2.9 %), to form a total of 3354 cells (129x26). The demographic data of the subjects are found in Table 1.

The result of the first cluster analysis of the A–F designs under the assumption that four-cluster solutions are the targets is shown in table 2. Both the ordinary Kappa and the weighted Kappa values are shown. As the classification of the four clusters in the reference data can be ranked with an ordinal scale from fast improvement to indifference in improvements a weighted Kappa may provide the most insightful result. In this comparison and with the Kappa criterion option E, every other week, has the best result, measured by the agreement with the reference data set. Options B and C are clearly inferior, and D and F are almost as good as E. It must be noted though, that this is a comparison of allocation into clusters, not a description of the trajectories themselves for the derived clusters.

**Table 2:** Distribution of subjects in clusters formed by the reference data (A) and the incomplete data sets (B–F)

	Reference, A, the full data set, 26 weekly measures				Kappa Agreement (Weighted Kappa)
	Cluster 1 “Fast improvers”	Cluster 2 “Normal improvers”	Cluster 3 “Slow improvers”	Cluster 4 “Indifferent”	
B, first 8 weeks					
Cluster 1	***N =*** **11**	*N =* 12	*N =* 0	*N =* 1	0.272 (0.548)
Cluster 2	*N =* 5	***N =*** **29**	*N =* 8	*N =* 0	
Cluster 3	*N =* 1	*N =* 9	***N =*** **9**	*N =* 13	
Cluster 4	*N =* 0	*N =* 13	*N =* 6	***N =*** **12**	
C, first 13 weeks					
Cluster 1	***N =*** **15**	*N =* 3	*N =* 0	*N =* 0	0.348 (0.611)
Cluster 2	*N =* 0	***N =*** **23**	*N =* 2	*N =* 5	
Cluster 3	*N =* 0	*N =* 33	***N =*** **7**	*N =* 0	
Cluster 4	*N =* 2	*N =* 4	*N =* 14	***N =*** **21**	
D, first 8 weeks + monthly thereafter					
Cluster1	***N =*** **14**	*N =* 4	*N =* 0	*N =* 0	0.618 (0.720)
Cluster 2	*N =* 1	***N =*** **52**	*N =* 8	*N =* 2	
Cluster 3	*N =* 0	*N =* 1	***N =*** **8**	*N =* 2	
Cluster 4	*N =* 2	*N =* 6	*N =* 7	***N =*** **22**	
E, every other week					
Cluster 1	***N =*** **16**	*N =* 15	*N =* 0	*N =* 0	0.642
Cluster 2	*N =* 1	***N =*** **45**	*N =* 7	*N =* 3	(0.823)
Cluster 3	*N =* 0	*N =* 1	***N =*** **13**	*N =* 0	
Cluster 4	*N =* 0	*N =* 2	*N =* 3	***N =*** **23**	
F, first 18 weeks					
Cluster 1	***N =*** **15**	*N =* 1	*N =* 1	*N =* 0	0.611 (0.708)
Cluster 2	*N =* 0	***N =*** **47**	*N =* 3	*N =* 1	
Cluster 3	*N =* 0	*N =* 7	***N =*** **8**	*N =* 1	
Cluster 4	*N =* 2	*N =* 8	*N =* 11	***N =*** **24**	

Figures in bold show number of subjects in B–F that are classified in clusters with similar trajectories as A

The agreement between A and B–F is estimated with the Kappa coefficient (both the raw Kappa and the weighted Kappa with quadratic weights).

**Fig. 1:**
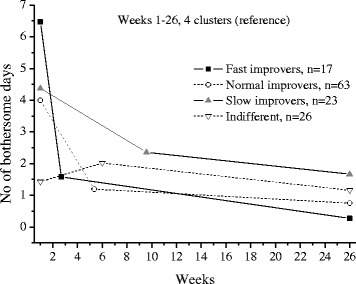
The cluster trajectories of option A

**Fig. 2:**
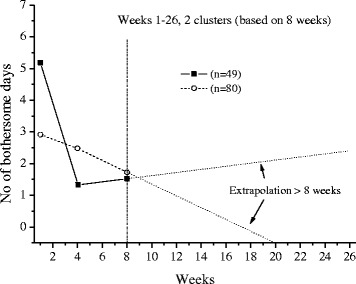
The cluster trajectories of option B

**Fig. 3:**
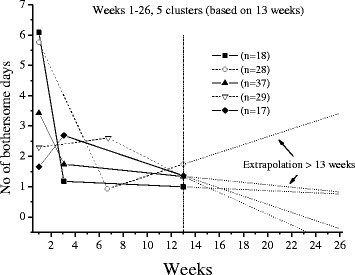
The cluster trajectories of option C

**Fig. 4:**
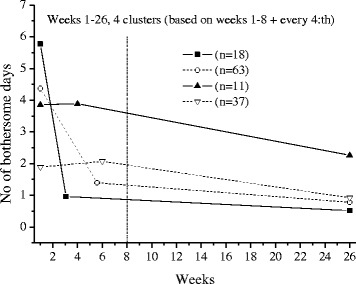
The cluster trajectories of option D

**Fig. 5:**
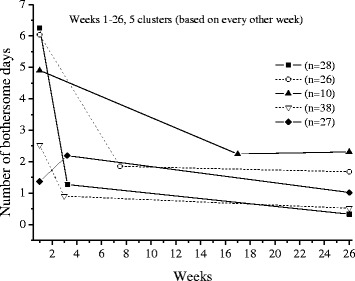
The cluster trajectories of option E

**Fig. 6:**
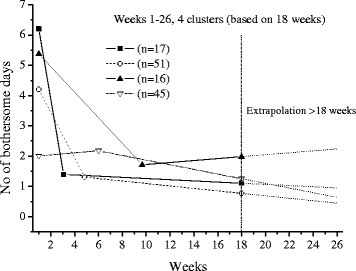
The cluster trajectories of option F

The extrapolations of the trajectories to cover 26 weeks, using the available data under each frequency option, are shown in Figs 1,2,3,4,5 and 6.

In the second evaluation of clustering and individual pain courses the optimal number of clusters differed between the frequency options. Option B resulted in two clusters, option C in five clusters, options D and F in four clusters and option E in five clusters. For the E option there was just a very small favour of 5 clusters over 4 clusters according to the Calinski-Harabasz criterion, an improvement with less than 0.7 % for 5 clusters compared with 4.

The trajectories reveal that the options B and C are not at all in line with the reference data. They both give a very inaccurate prediction of the development after weeks 8 and 13 weeks, apart from not being able to reproduce the number of clusters in the reference data. The measures of these two options most likely miss the knots, the trend breaks, which for a not ignorable number of subjects occur after week 8 and 13. Option D reproduces the assumed correct number of clusters but the trajectory for the slow improvers is not very accurate. This could be explained by the fact that the slow improvers have their knot after week 8 where option D has fewer measures. Option E does not the give the same number of clusters as the reference data, although the four cluster solution is almost obtained. It is mainly the cluster named “indifferent” that seems to be split up in two clusters. Finally, option F has the best profile of trajectories compared with the reference data. This option also covers most of the available time points.

**Fig. 7:**
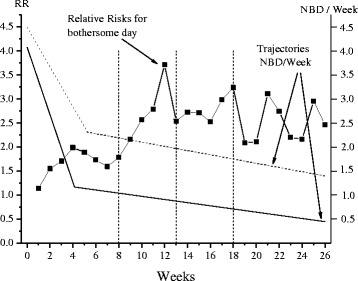
Relative Risks for experiencing a day with bothersome pain estimated from weekly SMS-answers separating subjects with pain of long duration (>30 days the previous year) from those with short duration (≤30 days the previous year). RR was estimated separately for each week, 1–26, with short duration as the reference category. Dotted vertical lines are shown to help the interpretation of results for measurements up to 8 weeks, 13 weeks and 18 weeks. The trajectories for the two groups are also shown, with short duration as the solid line

The relative risks for bothersomeness for the 26 weeks using previous duration as the explanatory factor are shown in Fig. 7. Experiencing > 30 days of pain the previous year lead to an increased risk of reporting bothersome pain during the study follow up. In the figure, the alternative use of the predetermined cut-offs at 8, 13 and 18 weeks are represented by dotted vertical lines. The RRs increase from week 1 to week 12 up to an RR higher than 3.5, then go down to a stabilized value approximately around 2.5. Using the options D and E give a more complete picture of the development of the Relative Risk for this data set, and option F gives almost as good a picture. Option B, 8 weeks, completely ignore the upward trend from week 8, and option C, 13 weeks, does not find the stable plateau after week 13.

## Discussion

In this study, we have used an existing dataset containing weekly pain measures from a completed observational study. The idea was to explore the minimal amount of information necessary to retain the overall result from the full dataset. By exploring the congruence between these “less than full” solutions with those obtained using the full dataset, empirical results may now inform a discussion of an optimal measuring frequency in a cohort of patients seeking care for LBP.

It is important to regard these results in light of the population and primary outcome studied. The developments of the subjects’ pain over time influence how measurement frequency may capture the pain trajectories. Studies of other variables with a more stationary behaviour over time may give rise to different considerations.

The result of clustering subjects showed that weekly measures for the first 8 weeks and only every 4 weeks thereafter as well as measures every other week, yield good to very good agreement with the reference data as regards allocation of individuals into clusters. However, in comparing trajectories, the best resemblance was found when using weekly data from the first 18 weeks. Therefore, selecting the optimal measuring frequency may be a matter of the aim of the study. If grouping (clustering) subjects recovering from a pain episode with similar profiles together, a short period of weekly measurements may be followed by monthly measurements. However, if the object is to study and estimate the “recovery trajectory” itself, it seems that using measurements every week for a limited amount of time is the best option. In this case 18 weeks resulted in the best performance, but the drawback is that this was the option that used the highest number of weekly measures of the options tested.

The result of using generalized linear models similarly indicate that options D (using the first 8 weekly and every fourth weekly measure thereafter) and E (using every other measure) most adequately describe both the initial change in RR and the steady state thereafter. Using only the first 13 weeks for analysis would completely miss the stabilized value that starts from around week 13. Using data up to and including week 18 would at least give a fairly good hint of a stabilized value in the risk estimation. Using only 8 weeks should be avoided with a population like this one. These results show that the existence of different individual courses of pain/bothersomeness might have a strong impact on the analytical model. If these trajectories are not adequately covered by the actual data the results might be even severely misleading.

The strengths of the study concern the quality and amount of data. It was collected with repeated text messages and had very good compliance (72 %) and short (1 week) recall. Further, as only those individuals with high compliance were selected, very few cells had to be imputed (2.9 %), rendering the estimates robust.

The main limitation of the study concerns the population under study, the fact that the observation started when the subjects sought care for LBP. Thus, the analysis and conclusions may only apply to subjects in similar situations. However, due to the fact that these subjects had clear trajectories (and not mere steady states) it was possible to compare the different measuring options in respect to their trajectories. Other studies with other types of variables (frequency of certain events, adherence to an intervention) may need to consider their outcomes in light of different measuring frequencies.

It should be noted that this was purely a data-driven approach, albeit one that originated in the reality of conducting clinical research. Therefore, the assumption was that a minimal measuring frequency would be the optimal frequency for compliance. The actual experiences of the subjects have not been illuminated in this regard. One could speculate that people would forget to answer when the measurements are further apart, that the weekly consistency appeals to a lot of people, and that monthly measures are more difficult to become routine events.

It is also important to point out that if measures are conducted wider apart, the recall period should be carefully considered. In order to maintain the validity of the measure, subjects should get the same question consistently. Thus, in this case with a measure pertaining to pain during the past week, if weekly measures became monthly, 3 weeks of each month would not be measured. This aspect needs to be considered in the equation of data quality and compliance.

## Conclusions

For the longitudinal study of a population consulting with LBP, subjects might be measured every week after a pain episode to capture the nature of their recovery. For a population with an initial phase of recovery followed by a steady state it is necessary to capture the first phase of a fast recovery with frequent weekly measures. For the second phase with a more steady-state condition, every 2 to 4 weeks might be adequate to capture the subgrouping properties, trajectory patterns and risk estimations. The clinical consequence needs to be tested in a prospective study, as the behavioural impact of measurements wider apart is not known. The burden to participants needs to be weighed against the need for frequent measures in light of the condition under scrutiny.

## Abbreviations

EQ-5D: Euro Qol 5-dimentions
LBP: Low back pain
NBD: Number of bothersome days
RR: Relative risk
SMS: Short message service (text message)
